# Alpha proteobacterial ancestry of the [Fe-Fe]-hydrogenases in anaerobic eukaryotes

**DOI:** 10.1186/s13062-016-0136-3

**Published:** 2016-07-30

**Authors:** Mauro Degli Esposti, Diego Cortez, Luis Lozano, Simon Rasmussen, Henrik Bjørn Nielsen, Esperanza Martinez Romero

**Affiliations:** 1 Italian Institute of Technology, Via Morego 30, 16136 Genoa, Italy; 2 Center for Genomic Sciences, UNAM Cuernavaca, Cuernavaca, Mexico; 3 Department of Systems Biology, Center for Biological Sequence Analysis, Technical University of Denmark, Kemitorvet, Building 208, 2800 Kongens Lyngby, Denmark

**Keywords:** Eukaryogenesis, Anaerobic metabolism, [FeFe]-hydrogenase, Mitochondria, Bioenergetics

## Abstract

Eukaryogenesis, a major transition in evolution of life, originated from the symbiogenic fusion of an archaea with a metabolically versatile bacterium. By general consensus, the latter organism belonged to α proteobacteria, subsequently evolving into the mitochondrial organelle of our cells. The consensus is based upon genetic and metabolic similarities between mitochondria and aerobic α proteobacteria but fails to explain the origin of several enzymes found in the mitochondria-derived organelles of anaerobic eukaryotes such as *Trichomonas* and *Entamoeba*. These enzymes are thought to derive from bacterial lineages other than α proteobacteria, e.g., *Clostridium* - an obligate anaerobe. [FeFe]-hydrogenase constitues the characteristic enzyme of this anaerobic metabolism and is present in different types also in *Entamoeba* and other anaerobic eukaryotes. Here we show that α proteobacteria derived from metagenomic studies possess both the cytosolic and organellar type of [FeFe]-hydrogenase, as well as all the proteins required for hydrogenase maturation. These organisms are related to cultivated members of the Rhodospirillales order previously suggested to be close relatives of mitochondrial ancestors. For the first time, our evidence supports an α proteobacterial ancestry for both the anaerobic and the aerobic metabolism of eukaryotes.

**Reviewers:** This article was reviewed by William Martin and Nick Lane, both suggested by the Authors.

**Electronic supplementary material:**

The online version of this article (doi:10.1186/s13062-016-0136-3) contains supplementary material, which is available to authorized users.

## Background

Mounting interest is arising on eukaryogenesis, a major transition in evolution which most likely originated from a symbiogenic event between an archaea and a metabolically versatile bacterium [1, 2]. The archaean partner of this symbiogenic event appears to be related to the Loki organisms recently discovered in deep ocean vents [2]. In contrast, the definition of the bacterial lineage for the other prokaryotic partner of eukaryogenesis remains unclear and controversial [2–9]. Recent analysis of major bioenergetic systems shared by bacteria and mitochondria suggests that a subset of extant α proteobacteria could be related to the ancestral lineage from which mitochondrial organelles of eukaryotic cells evolved [4, 8, 10]. However, biochemical and phylogenetic evidence emerged from the study of anaerobic eukaryotes seriously challenges the possibility that α proteobacterial organisms may be the ancestors also for the anaerobic metabolism of eukaryotes such as *Trichomonas* and *Entamoeba* [11, 12].

*Trichomonas*, *Entamoeba* and other eukaryotes adapted to anaerobic conditions possess Mitochondria Related Organelles (MRO [3, 12–14]) carrying out reactions of anaerobic metabolism that are typical of obligate anaerobes such as *Clostridium*, rather than α proteobacteria [12–19]. [FeFe]-hydrogenase constitutes the distinctive enzyme of this anaerobic metabolism and is present in different types [12–19], both in MRO and the cytosol of anaerobic eukaryotes such as *Mastigamoeba* [18]. The wide molecular variations of [FeFe]-hydrogenases [11, 12, 15–19] are present in many obligate anaerobes, in particular Clostridiales, Thermotogales and δ proteobacteria [12–19]; only a few facultative anaerobic α proteobacteria such as *Rhodospirillum rubrum* have similar hydrogenases, but not the maturases required for their biogenesis [13]. Consequently, it has been concluded that the eukaryotic types of this enzyme have been laterally acquired from bacterial lineages other than α proteobacteria, along various elements of anaerobic metabolism [11–13, 16–19]. Clearly, this conclusion undermines the hypothesis that a single α proteobacterium could have been the ancestor of both the anaerobic and the anaerobic metabolism of eukaryotes [1, 3, 6, 8].

Here we show that some α proteobacterial organisms found in metagenomic analysis of human microbiota [20] possess the two major types of eukaryotic [FeFe]-hydrogenases, as well as all genes needed for its assembly and maturation. Moreover, we report that relatives of photosynthetic *Rhodospirillum* [21] and methanotrophic α proteobacterial previously indicated as possible relatives of the mitochondrial ancestors [10, 22] possess a major type of eukaryotic [FeFe]-hydrogenases that could be the ancestor also of the hydrogenase-related Nar1 protein, which is involved in the biogenesis of Fe-S clusters of all eukaryotes [23]. Our findings thus rectify previous conclusions, strengthening the possibility that eukaryotes inherited also their anaerobic metabolism from a single α proteobacterial ancestor.

## Results and discussion

### Some α proteobacteria possess all the maturases for [FeFe]-hydrogenase

[FeFe]-hydrogenase requires the concerted action of three maturases, called *HydDEF* [13, 19, 24], for the complex assembly of its active site, the H cluster (see [25–27] for most recent advances on the subject). Previous studies concluded that the [FeFe]-hydrogenases present in α proteobacterial organisms such *Rhodospirillum rubrum* are probably inactive or play a different role because the genome of such organisms does not contain the *HydDEF* genes for maturases [13, 19]. However, we have found that bacteria closely related to *R. rubrum* possess all *Hyd* maturases and multiple genes for [FeFe]-hydrogenases (Table 1 and Additional file 1: Figure S1). These organisms include two species of the genera *Phaeospirillum* [21] and also *Pararhodospirillum* [28] (previously *Rhodospirillum photometricum* [29]). Two bacteria of the N_2_ fixing genus of *Pleomorphomonas*, classified among the Methylocystaceae family of methanotrophs [30], also possess the same set of proteins (Table 1). Moreover, we have found the presence of *Hyd* maturases in proteobacterial organisms of metagenomic origin that are classified among the α proteobacterial order of Rhodospirillales (Table 1), which have been assembled directly from the human gut microbiome [20]. The genes of these organisms were assembled from clustering of more than 396 human gut samples representing dense clusters and assembled to high quality draft genomes; their taxonomic classification was found to be robust at the genus level [20]. These clusters were confirmed and refined using a large collection of 2309 metagenomics samples. Given the read coverage and the quality of the assemblies, we expect the genomes to be nearly complete, with low probability that they may include genes from other sources [20]. In particular, *Acetobacter* sp. CAG:977 was identified in 14 samples and represented by as much as 4.5 % of the sequence reads in a stool sample while *Azospirillum* sp. CAG:239 was represented by as much as 11.6 % of the sequence reads in another stool sample. Other Rhodospirillales of human gut metagenomic origin containing *Hyd* maturases are *Azospirillum* sp. CAG:260 (Table 1) and *Acetobacter* sp. CAG:267 (see Additional file 1: Figure S1). Their proteins lie either in upstream or sister position with respect to the majority of the homologous maturase proteins of eukaryotes (see Additional file 1: Figure S1).

**Table 1:** Proteins of anaerobic metabolism in cultivated and uncultivated α proteobacteria

	Accession number of indicated proteins								
Taxonomic group	Clostridiales	α proteobacteria						Eukaryotes	
Genome	full genome	full genome	full genome	full genome	metagenome	metagenome	metagenome assembly, refined	full genome	full genome
Organism	*Eubacterium acidaminophilum*	*Pleomorphomonas oryzae*	*Phaeospirillum molischianum*	*Pararhodospirillum photometricum*	*Azospirillum CAG:260*	*Azospirillum CAG:239*	*Acetobacter MGS:igc0867* ^a^ *, formerly CAG:977*	*Entamoeba invadens*	*Trichomonas vaginalis*
Protein									
Fdsγ/NuoE-like	CAC39229, HymA	WP_051228661	CCG42368, HoxS	WP_041793924, HoxS	?	CDB53275	MH0030_GL0011535, CCZ22624	absent	AAV65813
Fdsβ/NuoF-like	CAC39230, HymB	WP_026791755			?	CDB53276	MH0276_GL0125578,CCZ22625	absent	AAV65812
Fdsα/NuoG-like, type M3 [FeFe]-hydrogenase	CAC39231, HymC	WP_026791754, WP_036838588	CCG42367, CCG40656	WP_051013609	?	CDB53277	MH0030_GL0047537, CCZ22626	M2 derivatives	M2 & M3 derivatives
type A [FeFe]-hydrogenase	AHM56319	absent	absent	absent	CDB39301	CDB53824	MH0143_GL0097789, CCZ21919	Yes, 5	absent
*HydE maturase*	WP_025434761	WP_051228492	WP_002729849	WP_041796462	CDB39229	CDB54134	MH0030_GL0015854, CCZ22110	absent	Yes, ≥ 2
*HydF maturase*	WP_025434763	WP_026790854	WP_002729850	WP_041793928	CDB40806	CDB53483	MH0030_GL0007678, CCZ21461	absent	Yes, ≥ 2
HydG maturase	WP_025434762	WP_026790852	WP_002729851	WP_041793926	CDB40807	CDB54364	MH0030_GL0045387, CCZ22171	absent	Yes, ≥ 2
PFO reductase	AHM55439	WP_036840581	CCG40004	CCG09362	CDB40895	?	MH0143_GL0088070, CCZ21865	Yes, ≥ 5	Yes, ≥ 1
Short ferredoxin	WP_025436274	WP_026790610.	CCG39997	CCG06940	CDB40908	CDB53367	MH0030_GL0000056, CCZ22365	Yes, ≥ 2	Yes, ≥ 3

^a^updated Meta Genomic Species with current CCZ accession number of formerly CAG:977

?not present in currently incomplete genome

We found that one gene for the long form of [FeFe]-hydrogenase in *Acetobacter* CAG:977 and *Azospirillum* sp. CAG:239 is associated with genes coding homologues of two redox subunits of respiratory complex I, which form the NADH-reacting module of the enzyme: NuoE and NuoF (Table 1, cf. [31–34]). The eukaryotic orthologs of these proteins are the 24 and 51 kDa subunits of mitochondrial complex I that are always coded by nuclear DNA, while the rest of complex I subunits is encoded by mtDNA in several protists [5, 32, 34]. The same proteins are found in the hydrogenosomes - a specialised form of MRO [19] - of *Trichomonas* [11] and *Sawyeria*, an anaerobic Heterolobosea [35], which lack complex I [19]. The gene cluster containing the long form of [FeFe]-hydrogenase and NuoEF homologues in the organisms identified from metagenomic analysis [20] resembles the *hymABC* operon described in Clostridiales [36, 37] (Table 1). In turn, this operon clearly derives from the Fds operon of NAD-dependent formate dehydrogenase [38], from which the NADH-reacting module of respiratory complex I originated [32, 34]. Of note, the genome of the abovementioned metagenomic α proteobacteria invariably contains the gene for Pyruvate:Ferredoxin Oxidoreductase (PFO, Table 1), a key enzyme in the anaerobic metabolism of Clostridiales [36, 37] and also anaerobic eukaryotes [19]. To summarise, we report the first example of α proteobacterial organisms that have all the *Hyd* maturases required for the assembly and function of [FeFe]-hydrogenase enzymes (Table 1), which often are present with multiple forms in their genome (Table 1).

### Uncultivated α proteobacteria possess both major forms of eukaryotic [FeFe]-hydrogenases

**Fig. 1:**
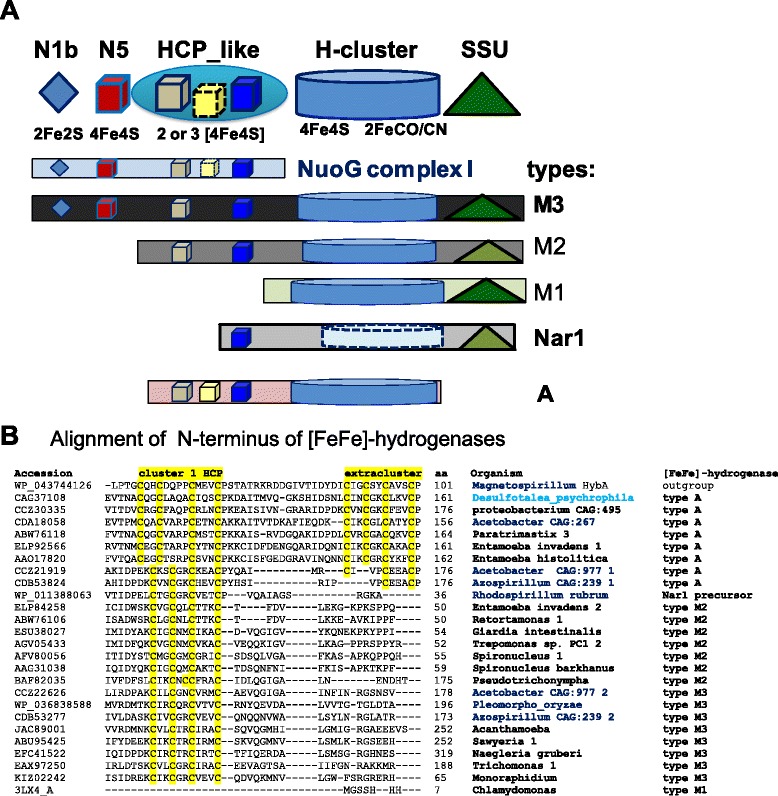
**a** The modular structure of various types of [FeFe]-hydrogenase is represented by different graphical symbols of the various domains labelled according to the CDD classification [50]. Cluster N5, which has a histidine ligand [31], is coloured in *red* to distinguish it from standard 4Fe4S clusters. The cartoon follows the nomenclature of Meyer [12] except for type A hydrogenase, which includes previous type M3a [12] and 6/8C subtypes [15]. The colour intensity of the Small Subunit (SSU) domain at the C-terminus [41] reflects the degree of conservation (Evalue) of the recognised domain [50]. **b** The N-terminal parts of various of various [FeFe]-hydrogenases were aligned first by using the COBALT feature of blast searches and then manually refined by giving maximal weight to the conservation of cysteine residues that could ligate FeS clusters [32]. The cysteine motifs binding FeS clusters are highlighted in *yellow*. The δ proteobacterium *Desulfotalea* is in *light blue* while α proteobacterial taxa are in *bold blue*; eukaryotic taxa are in *bold*

Anaerobic eukaryotes share diverse types of [FeFe]-hydrogenase with bacteria [11–19, 39]. The longest form of the enzyme, type M3 [12], is also the most common [40] and appears to be the progenitor of diverse eukaryotic variants [14]. At the N-terminus, type 3 proteins share three FeS domains with the NuoG subunit of complex I [11, 12]. Other types of [FeFe]-hydrogenase derive from the M3 type by differential loss of these FeS domains and progressive relaxation of sequence conservation in the Small Subunit (SSU) domain at the C-terminus [41], as summarised in the scheme of Fig. 1a. The cultivated α proteobacteria which we found to possess all *Hyd* maturases (Table 1) have one or more genes for type M3 [FeFe]-hydrogenase, which lie in intermediate branches of the phylogenetic tree of this common type of protein, often in sister position with respect to the monophyletic clade containing the eukaryotic homologues (see Additional file 1: Figure S2A). Conversely, uncultivated *Acetobacter* CAG:977 and *Azospirillum* sp. CAG:239 have not only type M3 [FeFe]-hydrogenases, but also another type of the enzyme which we have labelled type A (Fig. 1 and Table 1).

**Fig. 2:**
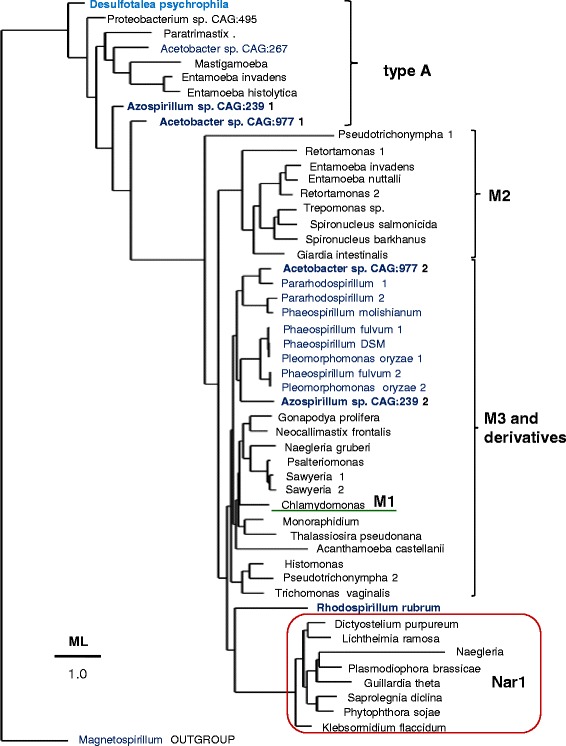
The tree illustrates consistent phylogenetic relationships between various types of [FeFe]-hydrogenases. The ML tree was obtained using the program PhyML [48] from a manually curated alignment of hydrogenase sequences retrieved by DeltaBLAST searches extended to uncultured organisms (cf. Additional file 1: Figure S2B). The known structure of algal [10] and bacterial [41] [FeFe]-hydrogenases has been used to implement the alignment refinement, using *Magnetospirillum HybA* protein sharing ferredoxin motifs with type A hydrogenases (accession: WP_043744126) as outgroup. The δ proteobacterium *Desulfotalea* is in *light blue* while α proteobacterial taxa are in *bold blue* as in Fig. 1b. All nodes have statistical support larger than 0.5. Note that two metagenomic organisms, *Acetobacter* CAG:977 and *Azospirillum* CAG:239, have representative proteins of both type A and type M3 [FeFe]-hydrogenases (cf. Table 1)

Type A [FeFe]-hydrogenase lacks the SSU domain at the C-terminus and characteristically shows an additional FeS cluster in the Hybrid Cluster Protein – like domain (HCP_like) at the N-terminus of the protein (Fig. 1). This structural feature derives from multicluster ferredoxins which are unrelated to type M3 [FeFe]-hydrogenases and therefore constitutes a major difference between [FeFe]-hydrogenases, making type A [FeFe]-hydrogenase a distinct enzyme (Table 1 and Fig. 2). A few δ proteobacteria such as *Desulfotalea psychrophila* possess ancestral versions of type A [FeFe]-hydrogenase, which is present also in the following eukaryotes: *Mastigamoeba* [18], *Entamoeba* [42] and *Paratrimastix* (formerly *Trimastix* [43]). In partial agreement with earlier reports [13, 18, 35], type A proteins occupy ancestral branches in phylogenetic trees of **[**FeFe]-hydrogenases, intermixing with related proteins from α proteobacterial organisms of metagenomic origin (Fig. 2, cf. Additional file 1: Figure S2B). Hence, we have found uncultivated α proteobacteria such as *Acetobacter* CAG:977 that possess both major types of the [FeFe]-hydrogenases that are present in eukaryotes, namely type A and type M3 (Table 1). Besides a few δ proteobacteria [12] and Clostridiales [15], this combination appears to occur in the eukaryotes, *Mastigamoeba* [18] and *Entamoeba* [42], which have cytosolic forms of the hydrogenase enzymes but have lost one or more maturases as in the truly amitochondriate *Monocercomonoides* [26] (cf. Table 1), a situation which is likely to derive from extreme metabolic simplification following loss of MRO [19].

In the course of this phylogenetic analysis, we have additionally found consistent clustering of the most divergent type of [FeFe]-hydrogenases, the Nar1 proteins [23], with the ancestral derivative of type M3 present in *R. rubrum* (Fig. 2 cf. Additional file 1: Figure S2B). This pattern could be discerned with strong bootstrap support after accurate sequence alignment of phylogenetically diverse proteins that revealed conserved elements shared with type M3 proteins from α proteobacteria (Fig. 2). Of note, Nar1 proteins were previously considered to form the basal branch in phylogenetic trees of [FeFe]-hydrogenases [17], presumably due to reliance on computer-assisted rather than manually cured sequence alignments for building such trees.

## Conclusions

**Fig. 3:**
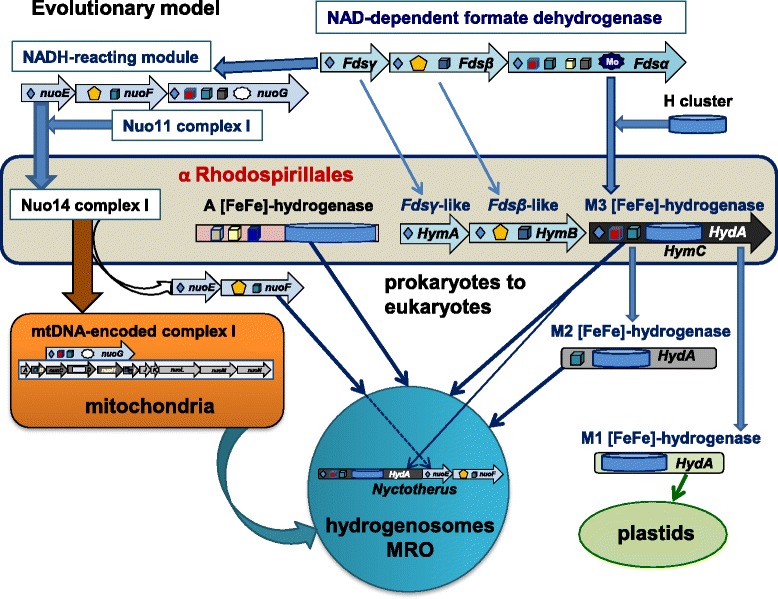
The cartoon illustrates the possible ancestry of [FeFe]-hydrogenases in eukaryotes from a Rhodospirillales organism related to metagenomic *Acetobacter* CAG:977. The Fds operon for NAD-dependent formate dehydrogenase [38] (*top part*) constitutes the common ancestor of both the NADH-reacting module of complex I – comprising subunits NuoEFG [34] – and the *HymABC* operon of clostridial [FeFe]-hydrogenases [36, 37]. In this operon, the N-terminal part of the original Fdsα subunit has merged with an ancestral Fe-S protein having the H cluster to form a prototypic type M3 [FeFe]-hydrogenase (HydA), which then has been transmitted to proto-eukaryotes in parallel to type A [FeFe]-hydrogenase (*central part*) and also the Nuo14 operon of complex I (*left part of the illustration*). The cartoon thus indicates that hydrogenosomes, anaerobic and aerobic mitochondria all derived from a common α proteobacterial ancestor, followed by specific gene loss and organelle relocation in different eukaryotes. Early after the initial symbiogenic event, the genes for the NuoEF subunits have been transferred from the ancestral bacterial genome to nuclear DNA and retained also in eukaryotic organisms containing hydrogenosomes such as *Trichomonas*. In the hydrogenosomes of the anaerobic ciliate *Nyctotherus*, the original NuoEF genes have been subsequently fused at the C-terminus of the *HydA* gene of [FeFe]-hydrogenases, thereby reproducing in a single protein the original *HymABC* operon that was present in the proteobacterial ancestor. However, the *HymB* subunit of this operon contains an additional 2Fe2S cluster at its N-terminus which is not present in the NuoF subunit of complex I and therefore distinguishes these two proteins from each other. No gene coding for a protein equivalent to *HymB* has been found in anaerobic eukaryotes, thereby suggesting that only the *HymC* prototype of eukaryotic M3 type [FeFe]-hydrogenase has been retained in the genome of the first eukaryote. Subsequent evolutionary divergence has produced eukaryotic lineages that have only derivatives of M3 type hydrogenase, while type A hydrogenase has been retained in the cytosol of a subset of flagellate protists that have highly deranged or no MRO [18, 26]. Note that the simplest derivative of type M3, type M1 is not present in mitochondria but in plastids [17], as shown in the *bottom right* of the illustration. Structural models for [FeFe]-hydrogenases are as shown in Fig. 1a. Other symbols are as follows (cf. [32]): *yellow pentagons*, flavin cofactors; *dark stars* with Mo inside, Mo-binding domains; *grey stars* without Mo, lost Mo-binding domains; *dark diamonds*, 2Fe2S clusters; *dark blue cubes*, 4Fe4S clusters; *red cubes*, 4Fe4S clusters with one histidine ligand, i.e., cluster N5 in NuoG; *grey cubes*, additional 4Fe4S clusters in Fdsα and the NuoG subunit of green complex I [33]; *yellow cubes*, additional 4Fe4S clusters in Fdsα and type A [FeFe]-hydrogenase

Contrary to established views that the anaerobic metabolism of eukaryotes might have been inherited through LGT phenomena from prokaryotes other than α proteobacteria [11, 12, 17, 18], we have shown here that a subset of α proteobacterial organisms of the Rhodospirillales order contain not only the two principal forms of eukaryotic **[**FeFe]-hydrogenases characteristic of this metabolism, but also the maturases required for the assembly of such enzymes (Table 1, Fig. 2 and Additional file 1: Figure S1). Our results therefore sustain the possibility that a single, metabolically versatile α proteobacterial organism might have been the ancestor of both the aerobic and anaerobic metabolism that is found in present day eukaryotes, in agreement with previous hypotheses [1, 3, 8, 44]. This simplifies the scenario of eukaryogenesis, since LGT events from organisms other than α proteobacteria [9, 11, 12, 18, 19] are no longer required. On the basis of our findings, we propose a model for the molecular evolution of **[**FeFe]-hydrogenases and the transmission of the associated anaerobic metabolism from an ancestral organism related to current Rhodospirillales to the proto-eukaryotic cell (Fig. 3). We are currently improving the metagenomic coverage of the organisms presented here and the isolation of individual cells for single cell sequencing to complete their genomes. Once the complete genome of *Acetobacter* CAG:977 will be obtained and analysed in depth, we will be able to further speculate on its relationships with the possible bacterial ancestor of both the aerobic and anaerobic metabolism of eukaryotes. In any case, it now appears to be clear that this ancestor was related to present day Rhodospirillales organisms.

## Methods

We have searched the latest version of the NCBI websites for proteins and genes in the NR and metagenomic databases using DeltaBLAST [45], as previously described [32, 46]. Sequence analysis was undertaken by first constructing refined alignments of phylogenetically wide selections of proteins, in some cases combining together proteins belonging to closely related families such as the SAM maturases (see Additional file 1: Figure S1) [10, 32, 33]. Initial sequence alignments were obtained with either MUSCLE or CLUSTALW by routinely using the BLOSUM62 substitution scale and then manually implemented by considering known 3D structures of representative proteins of the orthologous family. See the legend of Fig. 2 and see Additional file 1 for specific details. Such alignments were used to build reference trees with the MEGA 5.2 program [47] and subsequently refined using known domains and protein motifs from available 3D structures and iteratively implemented until the bootstrap values of nodes containing highly divergent proteins no longer increased. Phylogenetic analysis was then undertaken using, in general, the PhyML program [48] with the Maximal Likelihood (ML) approach and quantitative estimation of node strength as described [49]. Additional analysis was carried out with Neighbour-Joining (NJ) trees produced with the MEGA 5.2 program or the routine built into blast searches. Analysis of metagenomic data from human gut samples was undertaken as previously described [20].

## Reviewers’ comment

### Reviewer 1: William Martin

Reviewer summary

This is a very interesting paper reporting Fe-Fe hydrogenases from alphaproteobacterial genomes that branch as sisters to eukaryotic sequences. This further supports the view that the mitochondrial ancestor was a facultative anaerobe that was capable of mitochondrial type respiration and hydrogenosomal type fermentations. People will reinvestigate the topologies, but the findings reported here are of substantial importance for understanding the evolution of eukaryote physiology. The paper should be published as is.

Reviewer recommendations to authors

I don't see a lot that needs to be done. If people use different models with this data they will get different trees, probably, but the overall message sent by the paper is clear: gene diversity, gene transfer and pangenomes in prokaryotes are at least as important in the eukaryote gene origin issue as hair-splitting and often highly overparameterized single gene phylogenies. There was a nice paper by Ku et al. in PNAS last year talking about how pangenomes figure into the eukaryote gene origin issue. There was also a nice paper by Ku et al. in Nature last year that bears on these issues. Ettema and Gabaldon, the recent Nature papers cited the authors here, apparently still do not understand how LGT affects eukaryote gene origin issues. It is indeed complicated, as Martin Embley and I pointed out in 1998 (Nature) ans as I explained in some detail in 1999 (BioEssays). It is gratifying that microbiologists who understand some physiology, like Mauro Degli Esposti and colleagues, understand the issues that affect the origin of eukaryotic genes from organelle ancestors that live in a world of mosaic prokaryotic chromosomes.

Minor issues

The other referees will surely have a lot to complain about, I am very happy to see these findings.

MDE et al. response: *We thank the Reviewer for the very positive comments. In the revised manuscript, we have added the noted reference of Ku et al., Nature 2015 (new Ref. [8]).*

### Reviewer 2: Nick Lane

Reviewer summary

This is a good paper and seems to have been well done. I am not a phylogeneticist so I will defer review of the specific trees to other referees. However, the major conclusions are important, and the paper deserves publication, albeit with some recommended changes and clarifications.

This metagenomic study shows that genes for the two major eukaryotic types of [FeFe] hydrogenase are all present, along with genes for the required maturase enzymes, in the genomes of various alpha-proteobacteria related to Rhodospirillum rubrum. This finding is important in relation to the origin of the eukaryotic cell: was the bacterium that became the mitochondria a facultative anaerobe (in which case it was the ancestor of both hydrogenosomes and aerobic mitochondria) or was it an aerobic bacterium, in which case the [FeFe] hydrogenases were acquired independently on multiple occasions by lateral gene transfer from strictly anaerobic bacteria such as Clostridium? While there are several independent reasons to infer a single acquisition (see below), one problem in the past has been that not all the genes for [FeFe] hydrogenases and the maturases are present in alpha-proteobacteria.

This paper shows that in fact all the required genes are indeed present in close relatives of the facultatively anaerobic bacterium Rhodospirillum rubrum, and hence there is most likely to have been a common origin for aerobic and anaerobic metabolism in the mitochondria and hydrogenosomes. This is a pleasing finding that clarifies a controversial area and is wholly consistent with the predictions of the hydrogen hypothesis (Martin and Muller, Nature 1998), which explicitly predicts that the genes required for hydrogenesis derive from a facultatively anaerobic bacterium that was the common ancestor of both mitochondria and hydrogenosomes. This is also pleasing in that it is entirely consistent with bioenergetic considerations (e.g., Lane and Martin, Nature 2010) which also point to a singular origin of the eukaryotic cell in an endosymbiosis between an archaeon and a bacterium related to modern alpha-proteobacteria. Thus the paper supports the simple hypothesis that eukaryotes arose in a singular endosymbiosis between an archaeaon and a facultatively anaerobic bacterium.

Reviewer recommendations to authors

The major problem that I have with this paper relates to the evolutionary context. In my view the simple phrase 'mitochondria derive from alpha-proteobacteria' is not strictly correct, and could be seriously misleading. The problem is lateral gene transfer, not from bacteria into eukaryotes, but within bacteria (or between bacteria and archaea). The alpha-proteobacteria is a modern group, and they are well known to have evolved mechanisms that drive LGT. Given that the acquisition of mitochondria by an archaeal host cell occurred perhaps 1.5–2 billion years ago, it is not credible to talk about alpha-proteobacteria as if that modern group existed back then almost as it does now. And indeed from Fig. 2 it looks as if there has been some LGT of FeFE hydrogenase genes between different groups of bacteria.

We would therefore predict that many genes that were acquired with the ancestors of mitochondria should NOT branch with the modern alpha-proteobacteria, but rather with an assortment of modern groups. If they had all been acquired from a single endosymbiont then the key prediction is that they should all branch similarly from the base of the eukaryotes, not that they should all be found in modern alpha-proteobacteria. This seems to be the case (e.g., see Ku et al. PNAS 2015 and Ku et al. Nature 2015), although again this is by no means universally agreed. This context is entirely missing from the paper - the emphasis is wholly on the idea that genes for anaerobic metabolism should be found within modern alpha-proteobacteria, and a failure to find them would disprove a singular origin. That is not true. So while it is pleasing to find that they they are in fact common in the alpha-proteobacteria, this should not be couched as a make-or-break finding in the way that it is at several points in the paper.

I also think that Fig. 3 gives a misleading view of the hypothesis presented, and that the claim in the Conclusions for 'a new evolutionary model for [FeFe] hydrogenases…' is not correct and should refer instead far more explicitly to the hydrogen hypothesis, which predicted essentially all of this nearly two decades ago. My specific issue with Fig. 3 is that it makes it look as if the A [FeFe] hydrogenase and M3 [FeFe] hydrogenase were acquired separately in hydrogenosomes rather than via a mitochondrial ancestor which was similar to the alpha Rhodospiralles. The figure should make it clear that the first mitochondria were probably facultatively anaerobic with the same genes as the free-living bacteria, and that hydrogenosomes, anaerobic and aerobic mitochondria all derived from that common ancestor by specific gene loss within particular environments. This does not come across to me from that figure.

Minor issues

I have a few minor issues with some of the citations. For example in the first paragraph of the Background the statement 'originated from a symbiogenic event between an archaea and a metabolically versatile bacterium' is referenced to Sprang et al. and Williams et al. While both these references are appropriate for identifying the host cell as an archaeon, neither paper has anything specific to say about the endosymbiont being 'a metabolically versatile bacterium'. On the contrary - Sprang et al., by implying a phagocytic origin of mitochondria, actually obscures the nature of syntrophy in the endosymbiosis. The authors should cite here the hydrogen hypothesis (Martin and Muller Nature 1998); and on the nature of the host cell should also cite Sousa et al. Nature Microbiol 2016, which shows that Loki is hydrogen-dependent, also as predicted by the hydrogen hypothesis, and consistent with the results presented here.

Finally, as noted above, the paper omits any mention of other approaches to the origin of the eukaryotic cell, for example bioenergetic constraints (see e.g., Lane and Martin Nature 2010, and Lane Cold Spr Harb Pesp Biol 2014 for a discussion). These ideas have been critiqued by Booth and Doolittle PNAS 2015, and Lynch and Marinov PNAS 2015, and answered by Lane and Martin in PNAS 2015 and 2016. Other arguments in favour of a single origin of anaerobic and aerobic metabolism in mitochondria and hydrogenosomes include the very limited and repetitive metabolic repertoire of eukaryotes (equivalent to a single metabolically versatile bacterium; see e.g., Muller et al. Microbiol Moil Biol Rev 2012) and the ocean conditions during the Proterozoic when eukaryotes first arose (see e.g., various papers by Shields, Knoll and Canfield). The point is that there is a broader context than phylogenetics to framing the origin of the eukaryotic cell, and some of this literature should at least be mentioned.

That's all. I repeat, this is a good and interesting paper that is certainly worth publishing, but it would benefit from a broader discussion of the evolutionary context.

MDE et al. response: *We thank the Reviewer 2 for the positive comments and constructive criticisms. Our response is as follows*.

*Regarding further discussion on the broad evolutionary implications of our work, we now state at the end of the revised manuscript that …once the complete genome of Acetobacter CAG:977 will be obtained and analysed in depth, we will be able to further speculate on its relationships with the possible bacterial ancestor of both the aerobic and anaerobic metabolism of eukaryotes. In any case, it now appears to be clear that this ancestor was related to present day Rhodospirillales organisms*…

*With regard to the relationship between the ancestor of mitochondria and present day alpha proteobacteria, we appreciate the point raised by the Reviewer, which one of us has discussed amply in a paper that is about to be published (and available online - Degli Esposti M. Late Mitochondrial Acquisition, Really? Genome Biol Evol. 2016 Jul 3;8(6):2031-5. doi: 10.1093/gbe/evw130). However, the central problem that our paper is addressing is that previously [FeFe]-hydrogenases and their maturases were considered to be absent, or non functional, in alpha proteobacterial organisms, from which the ancestors of mitochondria have originated - according to the general consensus on the subject. For this reason we think it appropriate to maintain the relatively strong statements regarding the alpha proteobacterial ancestry of mitochondria, citing new references such as the suggested Ku et al. (Nature 2015) to further address the issue. With regard to Fig. 3, we have changed its presentation in the text as a model for the molecular evolution of [FeFe] hydrogenase, modified its graphical layout and changed its legend to better clarify that a single ancestral alpha proteobacterial organism might have transmitted both forms of hydrogenase to the protoeukaryotic cell, as suggested by the Reviewer.*

## Abbreviations

HCP_like, Hybrid Cluster Protein – like domain; LGT, lateral gene transfer; ML, maximum likelihood; MRO, Mitochondria Related Organelles; NJ, neighbour-joining; PFO, Pyruvate:Ferredoxin Oxidoreductase; SSU, Small Subunit

## Additional file

Additional file 1: Figure S1. shows the phylogenetic tree of SAM maturases HydE and HydF, while **Figure S2** shows the phylogenetic tree of type M3 [FeFe]-hydrogenase (A) and of all types of [FeFe]-hydrogenases. (PDF 415 kb)
