# Supplementary material for: Alpha proteobacterial ancestry of the [Fe-Fe]-hydrogenases in anaerobic eukaryotes
Source: Biol Direct. 2016 Jul 30;11:34. doi: 10.1186/s13062-016-0136-3 (PMC4967309; doi:10.1186/s13062-016-0136-3)
Supplement: Additional file 1: Figure S1. — shows the phylogenetic tree of SAM maturases HydE and HydF, while Figure S2 shows the phylogenetic tree of type M3 [FeFe]-hydrogenase (A) and of all types of [FeFe]-hydrogenases. (PDF 415 kb) [file 13062_2016_136_MOESM1_ESM.pdf]

Additional file 1

Supplementary Figures S1 and S2.

This file contains two supplementary figures. **Figure S1** shows the phylogenetic tree of SAM maturases HydE and HydF, while **Figure S2** shows the phylogenetic tree of type M3 [FeFe]-hydrogenase (A) and of all types of [FeFe]-hydrogenases (B).

Figure S1. Phylogenetic tree of SAM maturases for [FeFe]-hydrogenase show various α proteobacterial homologues. The ML tree was obtained using the program PhyML [47] from a manually curated alignment of sequences retrieved by DeltaBLAST searches extended to uncultured organisms. The known structure of bacterial maturases [25] has been used to implement alignment refinement. The *HydE* and *HydG* proteins lie in sister clades where α proteobacterial homologues are in ancestral branches. The related biotin synthase BioB occupies the basal clade. Nodes with statistical support larger than 0.5 [48] are marked by black dots. The tree was rooted using a distantly related protein from *Hyphomichrobium* (accession: WP_ 013215735) as outgroup. Eukaryotic taxa are in bold while α proteobacterial taxa are in **bold blue** with larger font. The dashed box indicates lack of monophyletic grouping of eukaryotic *HydE*, a situation confirmed using NJ trees (see also Ref [24]).

Figure S2. A. The NJ tree represents a simplified phylogenetic view of the long forms of [FeFe]-hydrogenase present in Thermotogales, Clostridiales, some proteobacteria and anaerobic eukaryotes. The NJ tree was obtained from the COBALT feature of blast [33] and complements that presented in Fig. 2 in taxonomic breath, even if it is restricted to type M3 hydrogenases. Note that the two β proteobacteria in the middle of the tree contain all *Hyd* maturases as for *Phaeospirillum* (Table 1), while the α proteobacteria *Rhodovulum* and *Rhodopseudomonas* do not contain such maturases (cf. [14]). **B.** NJ tree of all results obtained with a DeltaBLAST search using type M3 [FeFe]-hydrogenase domain of a *Nyctotherus* protein (accession: AAU14235) as a query. The search was extended to 1000 sequences including uncultured organisms from the following taxonomic groups. α proteobacteria: Rhodospirillales, Methylocystacee, *Magnetococcus, Rhizobium freirei* and *Microvirga lupini* for green complex I [33]; δ proteobacteria: *Desulfobacca*, *Desulfotalea psychrophila* and *Bilophila* for ancestral complex I and hydrogenase; eukaryotes, excluding higher plants and metazoans. The outgroup is represented by the highly divergent hydrogenase 7 from *Spironucleus* [16].

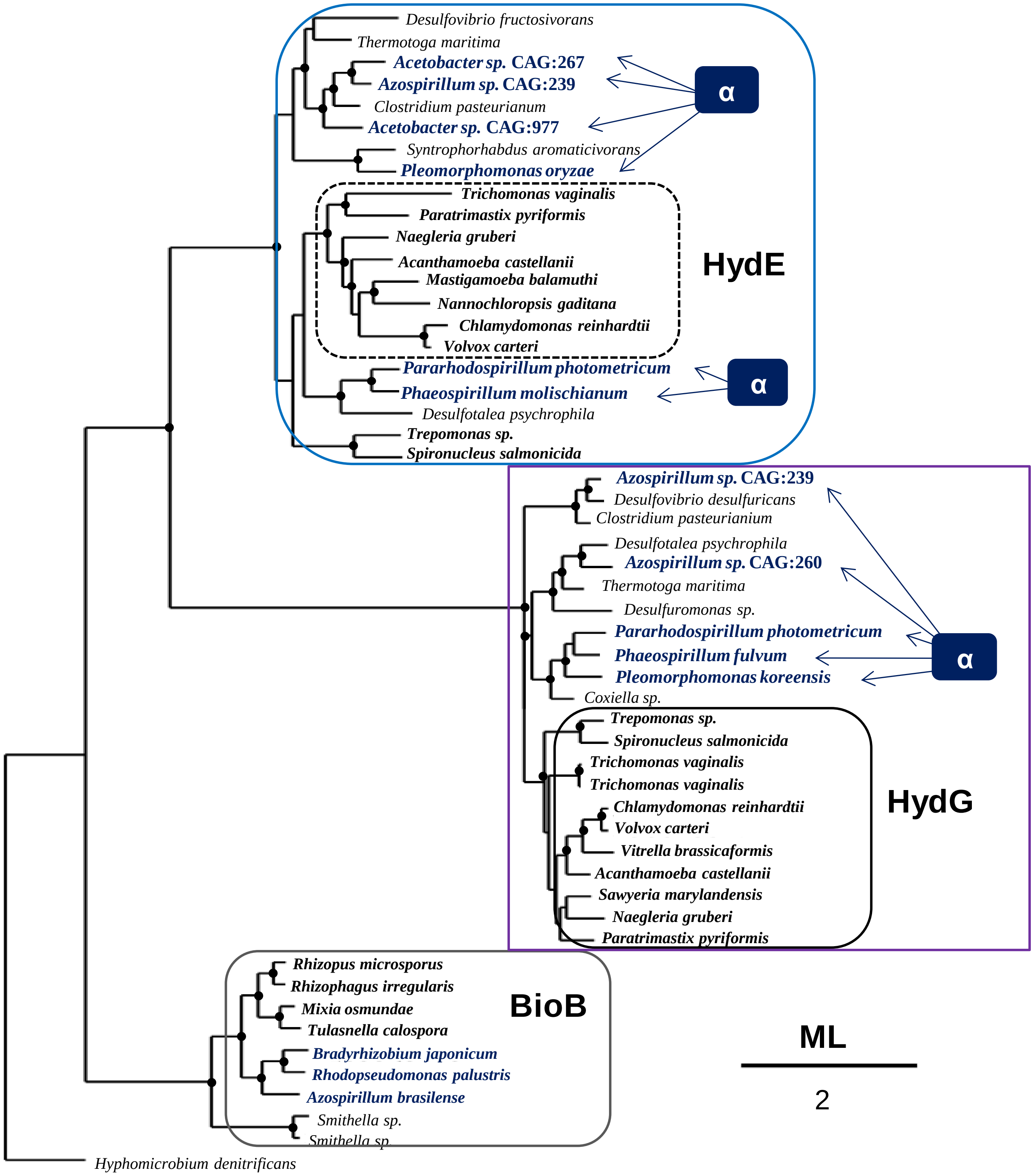
Desulfovibrio fructosivorans
Thermotoga maritima
Acetobacter sp. CAG:267
Azospirillum sp. CAG:239
Clostridium pasteurianum
Acetobacter sp. CAG:977
Syntrophorhabdus aromaticivorans
Pleomorphomonas oryzae
α
Trichomonas vaginalis
Paratrimastix pyriformis
Naegleria gruberi
Acanthamoeba castellanii
Mastigamoeba balamuthi
Nannochloropsis gaditana
Chlamydomonas reinhardtii
Volvox carteri
HydE
Pararhodospirillum photometricum
Phaeospirillum molischianum
α
Desulfotalea psychrophila
Trepomonas sp.
Spironucleus salmonicida
Azospirillum sp. CAG:239
Desulfovibrio desulfuricans
Clostridium pasteurianium
Desulfotalea psychrophila
Azospirillum sp. CAG:260
Thermotoga maritima
Desulfuromonas sp.
Pararhodospirillum photometricum
Phaeospirillum fulvum
Pleomorphomonas koreensis
α
Coxiella sp.
Trepomonas sp.
Spironucleus salmonicida
Trichomonas vaginalis
Trichomonas vaginalis
Chlamydomonas reinhardtii
Volvox carteri
Vitrella brassicaformis
Acanthamoeba castellanii
Sawyeria marylandensis
Naegleria gruberi
Paratrimastix pyriformis
HydG
Rhizopus microsporus
Rhizophagus irregularis
Mixia osmundae
Tulasnella calospora
BioB
Bradyrhizobium japonicum
Rhodopseudomonas palustris
Azospirillum brasilense
Smithella sp.
Smithella sp.
Hyphomicrobium denitrificans
ML
2

Figure S1

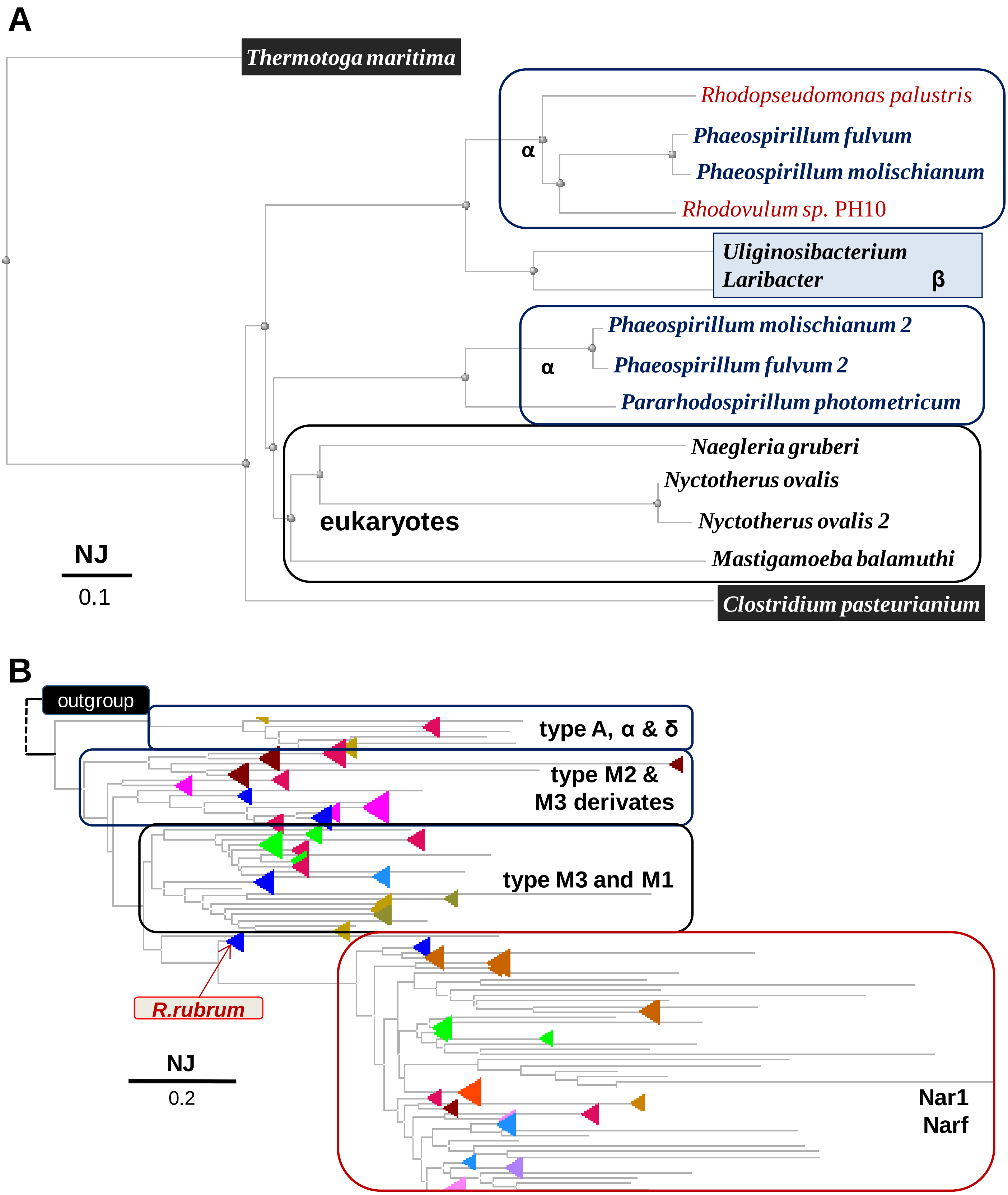
A
Thermotoga maritima
Rhodopseudomonas palustris
Phaeospirillum fulvum
Phaeospirillum molischianum
Rhodovulum sp. PH10
α
Uliginosibacterium
Laribacter
β
Phaeospirillum molischianum 2
Phaeospirillum fulvum 2
Pararhodospirillum photometricum
α
Naegleria gruberi
Nyctotherus ovalis
Nyctotherus ovalis 2
Mastigamoeba balamuthi
eukaryotes
Clostridium pasteurianium
NJ
0.1
B
outgroup
type A, α & δ
type M2 &
M3 derivates
type M3 and M1
R.rubrum
NJ
0.2
Nar1
Narf

Figure S2
